# Neuroprotective Effect of Tauroursodeoxycholic Acid on N-Methyl-D-Aspartate-Induced Retinal Ganglion Cell Degeneration

**DOI:** 10.1371/journal.pone.0137826

**Published:** 2015-09-17

**Authors:** Violeta Gómez-Vicente, Pedro Lax, Laura Fernández-Sánchez, Netxibeth Rondón, Gema Esquiva, Francisco Germain, Pedro de la Villa, Nicolás Cuenca

**Affiliations:** 1 Departamento de Óptica, Farmacología y Anatomía, Universidad de Alicante, Alicante, Spain; 2 Departamento de Fisiología, Genética y Microbiología, Universidad de Alicante, Alicante, Spain; 3 Departamento de Biología de Sistemas, Universidad de Alcalá, Alcalá de Henares, Spain; Dalhousie University, CANADA

## Abstract

Retinal ganglion cell degeneration underlies the pathophysiology of diseases affecting the retina and optic nerve. Several studies have previously evidenced the anti-apoptotic properties of the bile constituent, tauroursodeoxycholic acid, in diverse models of photoreceptor degeneration. The aim of this study was to investigate the effects of systemic administration of tauroursodeoxycholic acid on N-methyl-D-aspartate (NMDA)-induced damage in the rat retina using a functional and morphological approach. Tauroursodeoxycholic acid was administered intraperitoneally before and after intravitreal injection of NMDA. Three days after insult, full-field electroretinograms showed reductions in the amplitudes of the positive and negative-scotopic threshold responses, scotopic a- and b-waves and oscillatory potentials. Quantitative morphological evaluation of whole-mount retinas demonstrated a reduction in the density of retinal ganglion cells. Systemic administration of tauroursodeoxycholic acid attenuated the functional impairment induced by NMDA, which correlated with a higher retinal ganglion cell density. Our findings sustain the efficacy of tauroursodeoxycholic acid administration *in vivo*, suggesting it would be a good candidate for the pharmacological treatment of degenerative diseases coursing with retinal ganglion cell loss.

## Introduction

Retinal ganglion cells (RGCs) are the output neurons of the retina, whose axons converge at the optic disk to form the optic nerve. RGCs collect and integrate visual information from second-order neurons and then transmit electrical impulses from the retina to the brain. Loss of RGCs is a hallmark of a number of retinal or optic nerve diseases such as diabetic retinopathy, retinal ischemia, glaucoma, or Leber hereditary optic neuropathy [1–3]. While in fish and amphibians RGC neurogenesis may be extended into adulthood, in mammals this process is restricted to the period of embryonic/neonatal retinal development (reviewed in [4]), meaning these post-mitotic neurons are irreplaceable in the mature, terminally differentiated, retina. Therefore, RGC loss implies progressive and permanent vision impairment. In this context, the discovery of compounds that enhance RGC survival might be of therapeutic interest.

Bear bile has been used in ancient Chinese medicine for the improvement of visual acuity, but it has not been until recently that several investigations have documented the anti-apoptotic properties of the bile constituent tauroursodeoxycholic acid (TUDCA) in rodent models of photoreceptor degeneration, including light-induced retinal damage [5], retinitis pigmentosa [6–9] experimental retinal detachment [10] and Leber congenital amaurosis [11]. Photoreceptor loss was significantly delayed by TUDCA in these retinal disease models, simultaneously with a general improvement of retinal morphology and function. Nevertheless, studies reporting the anti-apoptotic effect of TUDCA on visual disorders affecting retinal neurons other than photoreceptors are scarce. Boatright and colleagues reported a neuroprotective effect of TUDCA on RGC degeneration following optic nerve transection in the mouse [12], but to date the efficacy of TUDCA has not been tested in any other RGC death models or animal species. In the present study, we address this issue using electroretinographical and histological techniques to evaluate the neuroprotective potential of TUDCA against N-methyl-D-aspartate (NMDA)-induced retinal injury *in vivo*. Although perhaps more relevant from a physiological point of view, genetic models of RGC degeneration, such as the DBA/2J mouse, take considerably long to develop and display high between-animal variability regarding disease progression [13–15]. Intravitreal injection of NMDA, in contrast, represents an acute animal model of excitotoxicity, reasonably convenient for drug screening and efficacy studies, as it causes reproducible and fast RGC death in rodents [16–19]. Excessive stimulation of NMDA receptors, one of the three ionotropic glutamate receptor subtypes expressed in inner retinal cells, induces a series of events such as perturbation of Na^+^/K^+^ homeostasis, Ca^2+^ overload, mitochondrial dysfunction and oxidative stress [17,20–22], that ultimately lead to cell death.

After performing a detailed, quantitative analysis of RGC distribution and function following excitotoxic insult, we confirm here that systemic administration of TUDCA enhances RGC survival. Clinical trials of TUDCA are currently active or in recruiting phase for various pathologies, including cystic fibrosis, cholestasis, diabetes/obesity and amyotrophic lateral sclerosis (NIH Clinicaltrials.gov NCT00004441, NCT01829698, NCT00771901, NCT00877604). Our results provide a proof of principle of the efficacy of TUDCA as a neuroprotective factor for RGC, paving the way for clinical trials on glaucoma patients and other degenerative diseases coursing with RGC death.

## Materials and Methods

### Animals and treatments

Experimental procedures were carried out in strict accordance with the current regulations for the use of laboratory animals (ARVO statement for the use of animals in ophthalmic and visual research and European Directive 2010/63/UE) and all efforts were made to minimize animal suffering and numbers. The protocol was approved by the University of Alicante Research Ethics Committee (permit number #UA-2013-07-22). Sprague-Dawley rats, obtained from Harlan laboratories (Indianapolis, IN, USA), were used in this study. The animals were bred at the University of Alicante animal facilities and reared in an artificial 12-h light/dark cycle with food and water *ad libitum*.

Tauroursodeoxycholic acid (TUDCA; Calbiochem, Merck Millipore, Darmstadt, Germany) was dissolved in phosphate-buffered saline solution, pH 7.4, and sterile-filtered prior to administration. Adult (12–16 weeks) rats received a daily intraperitoneal dose of TUDCA (500 mg/kg) or vehicle (phosphate-buffered saline) for 6 days.

### Electroretinogram (ERG)

**Fig 1:**
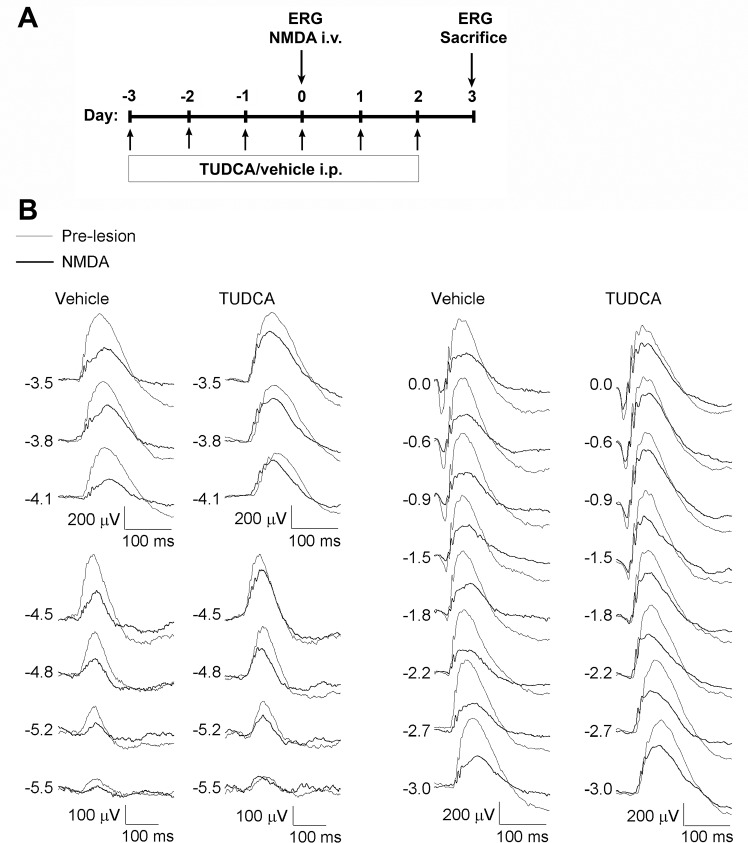
Effect of NMDA and TUDCA on the rat full-field ERG. (A) Experimental timeline indicating the days of intraperitoneal injection with TUDCA or vehicle, ERGs and intravitreal delivery of NMDA. (B) Representative scotopic ERG waveforms performed before (thin traces) and after (bold traces) NMDA-induced retinal lesion in rats treated with TUDCA or vehicle. Units on the left indicate input flash intensities in log cd·s/m^2^.

ERG recordings were first performed on the fourth day of treatment with TUDCA or vehicle, immediately before intravitreal delivery of NMDA. ERG responses were again evaluated on the seventh day, i.e. three days after inducing retinal damage (timeline is shown in Fig 1A). In each case recordings were performed at least 24 h after TUDCA or vehicle administration, to avoid the influence of stress or treatment-derived acute effects on ERG responses.

Dark-adapted (12 h) rats were anaesthetized with an intraperitoneal injection of ketamine (100 mg/kg) and xylazine (4 mg/kg) solution, and were maintained on a thermal blanket at 38°C for the entire procedure. Pupils were dilated by topical application of 1% tropicamide (Alcon Cusí, Barcelona, Spain) and a drop of 0.2% polyacrylic acid carbomer (Viscotears; Novartis, Barcelona, Spain) was instilled on each eye to prevent corneal dehydration and to optimize electrical contact with the recording electrodes, which were DTL fiber electrodes with a silver-coated nylon conductive yarn (X-Static; Sauquoit Industries, Scranton, PA, USA). The reference electrode was a 25-gauge platinum needle inserted under the scalp, between the eyes, and the ground electrode was placed in the mouth. Animal handling and preparation was done under dim red light, then anaesthetized rats were placed on a Faraday cage and all experiments were performed in absolute darkness.

Scotopic flash-induced ERG responses were recorded simultaneously from both eyes in response to light stimuli produced with a Ganzfeld stimulator. Light stimuli were presented for 10 ms at 15 different increasing intensities ranging from -5.5 to 0 log cd·s/m^2^. 3 to 10 consecutive recordings, 500 ms in duration, were averaged for each light presentation. Scotopic threshold responses (STR) were obtained for flash intensities ranging from -5.5 to -4.5 log cd·s/m^2^. The interval between light flashes was 10 s for dim flashes (-5.5 to -1.5 log cd·s/m^2^) and up to 20 s for the highest intensity (-0.9 to 0 log cd·s/m^2^). ERG signals were amplified and band-pass filtered (0.1–1000 Hz) using a commercial amplifier (DAM 50; World Precision Instruments, Aston, United Kingdom), and digitalized at 4 kHz with a PowerLab acquisition device (ADInstruments; Oxfordshire, United Kingdom). To visualize oscillatory potentials, the signal recorded was filtered between 100 and 1000 Hz. The amplitude of the pSTR was measured from the baseline to the peak of the pSTR (∼115 ms after the stimulus); the amplitude of the nSTR was measured from the baseline to the trough of the nSTR (∼220 ms after the stimulus); the amplitude of the a-wave was measured from the baseline to the trough of the a-wave (∼15 ms after the stimulus); and the amplitude of the b-wave was measured from the trough of the a-wave to the peak of the b-wave (∼50 ms after the stimulus). For oscillatory potentials the maximum peak-to-trough amplitude was considered.

### Intravitreal injection

Using a 30-gauge needle an initial puncture was made in the dorso-temporal sclera, about 1 mm from the sclerocorneal limbus. A 33-gauge needle, coupled to a Hamilton syringe, was then introduced to the vitreous cavity and 3 μl of 20 mM NMDA (60 nmol; Sigma, St. Quentin Fallavier, France) were injected in both eyes of each animal. The cannula was left in place for one minute and then slowly withdrawn. The animals were housed in individual cages and allowed to recover from anaesthesia on a warm water pad. Ocular lubrication was provided. Animals with lens damage or vitreal haemorrhage were excluded from the study.

### Immunohistochemistry

At the end of the treatment, animals were euthanized by cervical dislocation under deep anaesthesia and the retinal tissue was harvested and processed for immunohistochemistry. Previously to eye enucleation, a suture was placed on the superior pole of each eye to maintain retinal orientation. Enucleated eyes were fixed in freshly made 4% (w/v) paraformaldehyde, in 0.1M phosphate buffer pH 7.4, for 1 h at room temperature, and washed several times with phosphate buffer. Then, the cornea and lens and vitreous body were carefully removed and the retina was dissected out. The retinas were incubated for 72 h at 4°C with goat polyclonal anti-Brn3a antibody (1:500; #sc-31984L, Santa Cruz Biotechnology Inc., Santa Cruz, CA, USA) and rabbit polyclonal anti-RBPMS antibody (1:10000; a generous gift from Dr. Nicholas Brecha), diluted in 0.1M phosphate buffer containing 1% (v/v) Triton X-100 (Sigma). After several washes with phosphate buffer, the retinas were incubated for 2 h at room temperature with a cocktail of secondary antibodies, Alexa Fluor 488 donkey anti-goat IgG (1:500; Molecular Probes®, Eugene, OR, USA) and Alexa Fluor 555 donkey anti-rabbit IgG (1:500; Molecular Probes®). Then, the retinas were washed, flat-mounted on glass slides with the vitreous side up, coverslipped with anti-fading mounting medium (Citifluor Ltd., London, United Kingdom) and sealed with nail polish.

### Confocal microscopy and quantification of surviving RGCs

Using a laser-scanning confocal microscope (TCS SP2, Leica Microsystems, Wetzlar, Germany), serial horizontal xy-sections, 4 μm in depth, were acquired in the z-axis with a 20X objective along the dorsal-ventral and nasal-temporal axes of the retina. Double positive Brn3a/RBPMS cells in the ganglion cell layer were scored in maximal confocal projections at 16 regions of interest (four areas per retinal quadrant at different eccentricities, 1, 2, 3 and 4 mm from the optic disc; measuring 400 x 400 μm^2^ each). Mean density (number of cells per mm^2^) values were calculated for all eccentricities as well as over the whole retina. A total of 6 retinas per experimental group (untreated, vehicle+NMDA or TUDCA+NMDA) were analyzed.

### Statistical analysis

Statistical analyses were performed using SPSS 18.0 software (IBM Armonk, NY, USA). A two-way repeated measures ANOVA was performed to evaluate the effects of the treatment (vehicle vs. TUDCA) on ERG responses throughout the experimental stages (before and after inducing retinal damage with NMDA). A two-way ANOVA was performed to evaluate differences in the mean density of RGCs between the three experimental groups (untreated, vehicle+NMDA or TUDCA+ NMDA) at the distinct eccentricities. When a 0.05 level of significance was found, post-hoc pairwise comparisons using Bonferroni’s test were made. Normal distributions and homogeneity of variance were found for all analyzed categories. P values less than 0.05 were considered statistically significant. Data were plotted as the mean ± standard error of the media (SEM).

## Results

### Intravitreal injection of NMDA reduces retinal responsiveness

**Fig 2:**
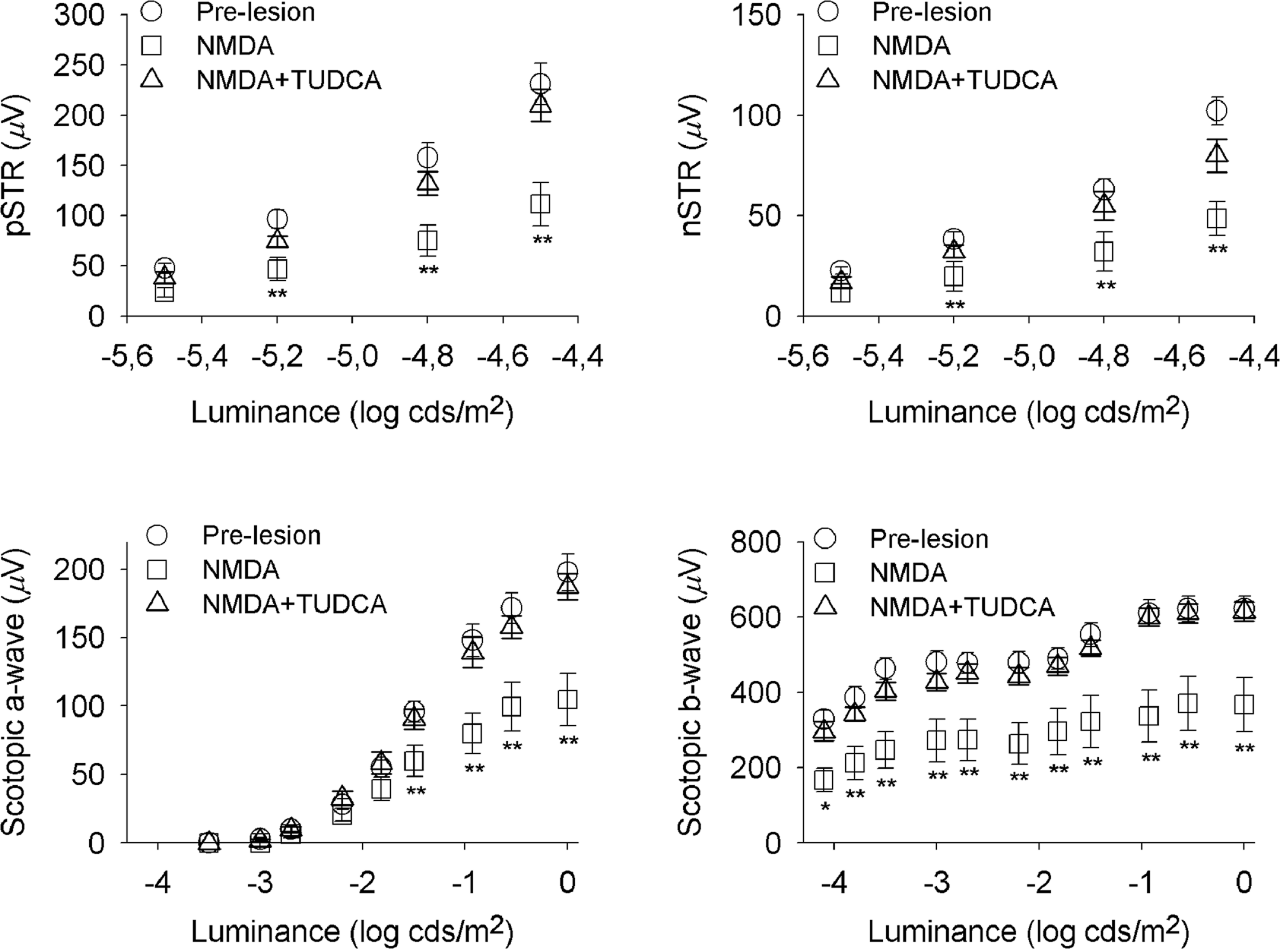
Effect of NMDA and TUDCA on the ERG intensity-response functions. The graph represents mixed scotopic ERG amplitude (mean ± SEM) versus stimulus intensity previous to retinal damage (pre-lesion, circles, n = 11), and after NMDA-induced lesion in rats either treated with vehicle (NMDA, squares, n = 5) or TUDCA (NMDA+TUDCA, triangles, n = 6). Scotopic pSTRs, nSTRs, a-waves and b-waves recorded after retinal insult in TUDCA-treated rats reached higher values than those obtained in vehicle-administered animals. Asterisks indicate statistical significance (ANOVA, Bonferroni’s test), *P<0.05, **P<0.01.

**Fig 3:**
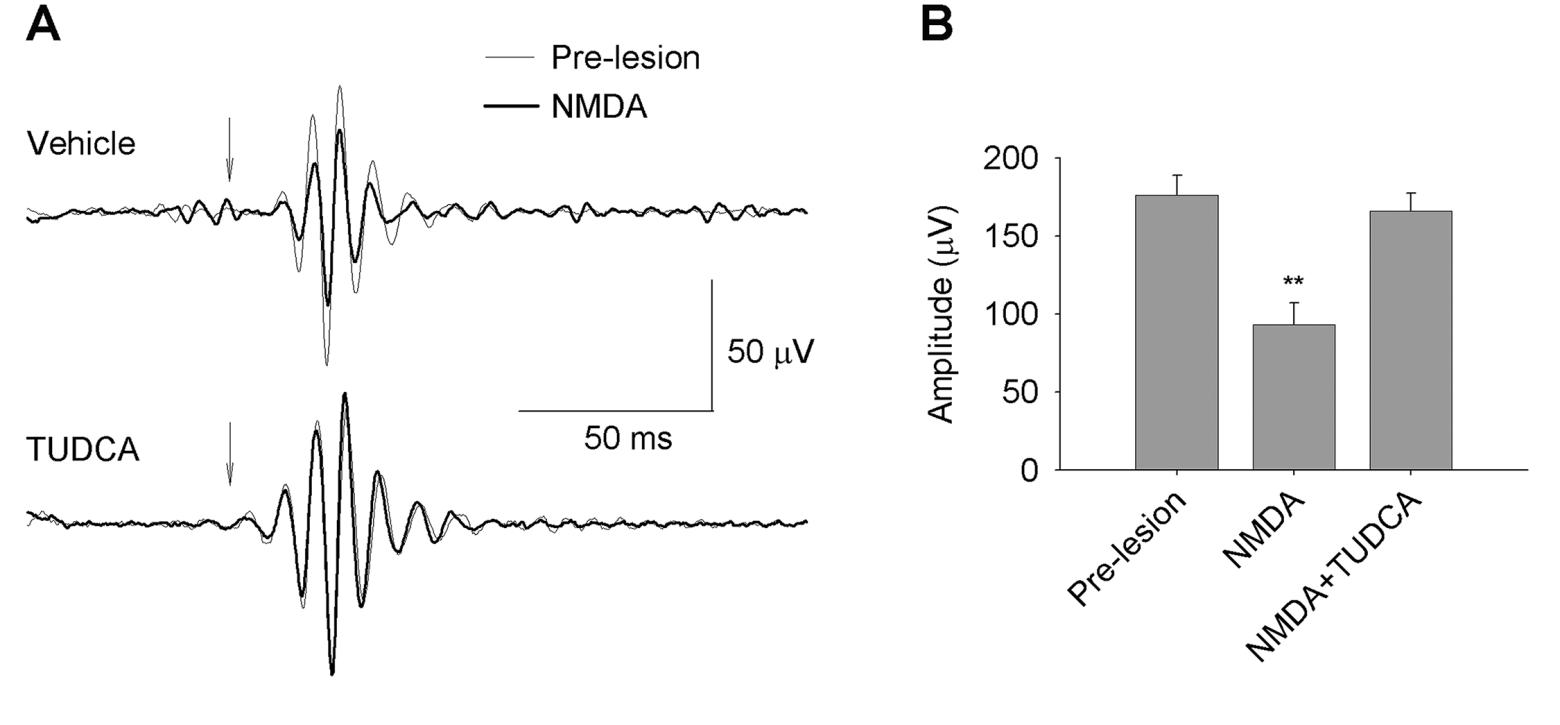
Effect of NMDA and TUDCA on the ERG OPs. (A) Representative examples of filtered OP traces from scotopic ERGs recorded before (thin traces) and after (bold traces) NMDA-induced retinal damage, in vehicle- (upper graph) or TUDCA-treated (lower graph) rats, in response to a 1 cd·s/m^2^ stimulus (arrow). (B) Amplitude (mean ± SEM) of maximum OPs before retinal damage (pre-lesion, n = 11), and after NMDA-induced lesion in rats either treated with vehicle (NMDA, n = 5) or TUDCA (NMDA+TUDCA, n = 6). Asterisks indicate statistical significance (ANOVA, Bonferroni’s test) for pre-lesion *vs*. NMDA, and NMDA *vs*. NMDA+TUDCA groups, **P<0.01. No significant differences were found when comparing pre-lesion *vs*. NMDA+TUDCA groups.

In order to evaluate retinal functionality, we performed full-field ERG recording in dark-adapted conditions, before and after NMDA-induced retinal lesion (Fig 1A). Several reports suggest that, in rodents, the ERG response to a very dim light stimulus, near the scotopic threshold, depends on inner retina function, specifically on RGCs [23–26]. Accordingly, this ERG response has been called the STR and consists of a positive potential followed by a negative component, known as the pSTR and nSTR, respectively. Representative scotopic full-field flash responses to stimulus of increasing intensity are shown in Fig 1B. STR components were evaluated for flash intensities ranging from -5.5 to -4.5 log cd·s/m^2^. With increasing stimulus intensity both, the pSTR and nSTR, grew in amplitude. The positive and negative components had their peak amplitudes at ∼115 ms and ∼220 ms, respectively. At higher stimulus intensities the a- and b-waves dominated the ERG, with peak amplitudes at ∼15 ms and ∼50 ms, respectively. The relationship between stimulus intensity and ERG amplitude is represented in Fig 2. As it can be observed in Figs 1B and 2, intravitreal injection of NMDA caused a significant reduction in the mean amplitude of all these parameters: pSTR (52% less), nSTR (49% less), scotopic a-wave (38% less) and scotopic b-wave (42% less) (ANOVA, Bonferroni’s test, P < 0.01 in all cases). Although, NMDA-mediated toxicity did not affect the implicit time of pSRT or nSTR, it caused a significant increase in the implicit time of a- and b-wave (7% more in both cases), as compared to untreated control rats (ANOVA, Bonferroni’s test, P < 0.01 in both cases). In addition, scotopic oscillatory potentials (OP), which reflect light-induced activity in the inner retina [27], were recorded at 0 log cd·s/m^2^ before and after NMDA-induced damage (Fig 3). Pharmacological studies indicate that both, RGCs and amacrine cells are involved in the events underlying OPs [27,28]. OPs amplitude in rats was significantly higher (ANOVA, Bonferroni’s test, P < 0.01) before NMDA-induced lesion (amplitude: 176 ± 13 μV; latency: 30.9 ± 0.4 ms) than after the damage (amplitude: 93 ± 14 μV; latency: 32.9 ± 1.2 ms). Differences in the implicit time were not significant.

### TUDCA attenuates retinal function decline induced by NMDA

To test the neuroprotective effect of TUDCA on retinal function, full-field ERG recording was performed in parallel on a group of rats that received a daily intraperitoneal dose of TUDCA three days prior to NDMA administration, and until their sacrifice (Fig 1A). ERG responsiveness was less deteriorated by NMDA in TUDCA-treated than in vehicle-treated animals (Figs 1B and 2). Although TUDCA did not completely prevent the fall in retinal responsiveness upon NMDA injection, it reduced the damage considerably. pSTR and nSTR amplitudes recorded after NMDA injection were significantly higher in TUDCA-treated rats (72% and 62% more, respectively), as compared to vehicle-treated animals (ANOVA, Bonferroni’s test, P < 0.01 for pSTR and P < 0.05 for nSTR). TUDCA treatment also promoted in NMDA-injected animals scotopic a- and b-wave amplitudes significantly higher (61% and 66% more, respectively) than those observed in vehicle-administered control rats (ANOVA, Bonferroni’s test, P < 0.01 in both cases). The effect of TUDCA on the a-wave implicit time in NMDA-injected rats was negligible, however, it promoted a significant reduction on the b-wave implicit time observed in NMDA-damaged rats (2.3% less; ANOVA, Bonferroni’s test, P < 0.01). Furthermore, TUDCA treatment significantly reduced the deleterious effect of NMDA on the scotopic OPs, as shown in Fig 3. Whereas TUDCA administration did not completely abolish the decrease in the OP amplitude caused by NMDA, scotopic OP amplitudes after NMDA-induced lesion were significantly higher (ANOVA, Bonferroni’s test, P < 0.01) in TUDCA-treated than in vehicle-treated rats (78% more).

### RGC numbers decrease following intravitreal NMDA delivery

**Fig 4:**
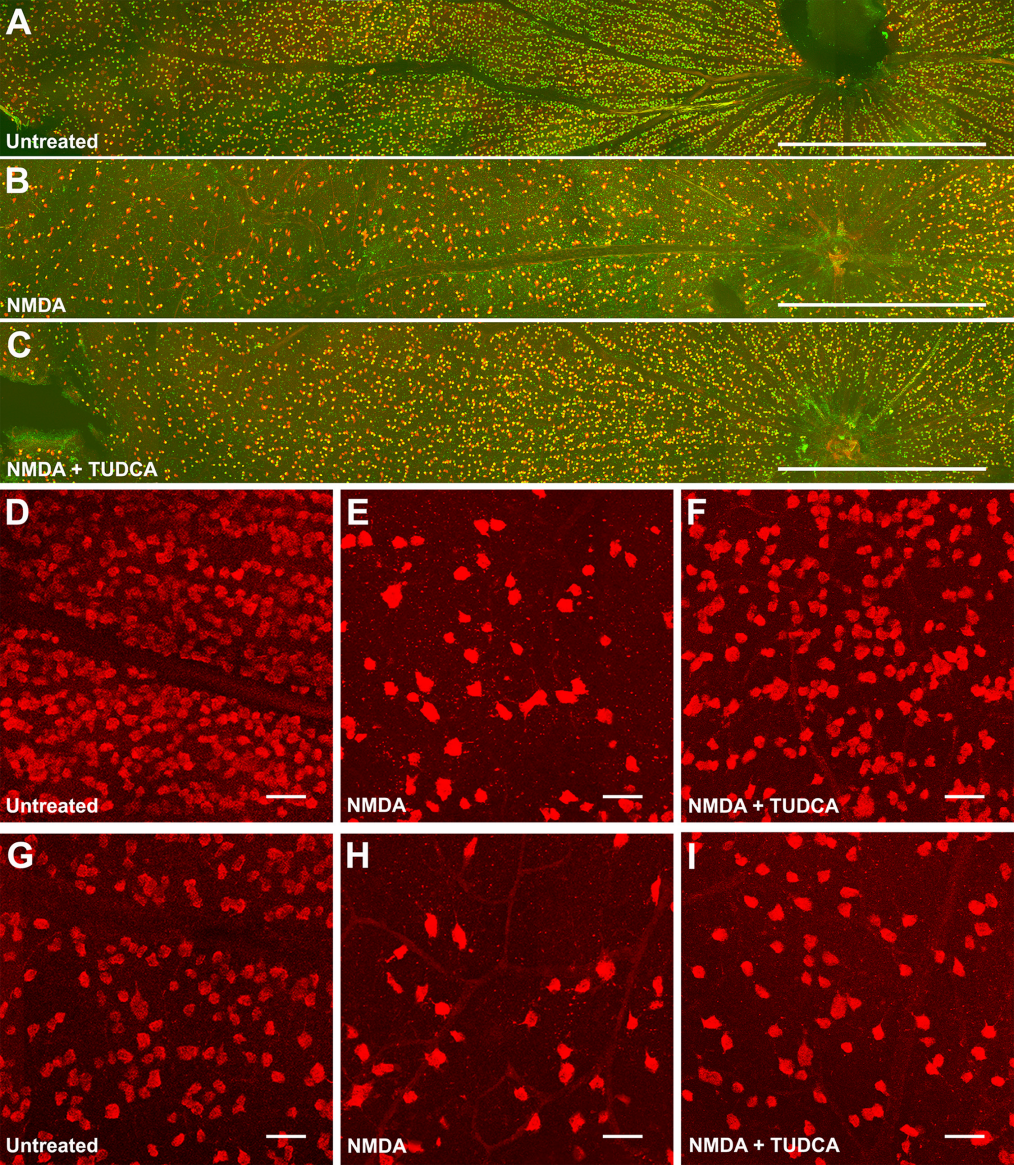
Immunohistochemical analysis of RGCs after NMDA-induced damage in the presence or absence of TUDCA. Confocal images of whole-mounted retinas labelled with the RGC markers Brn3a (green) and RBPMS (red). A representative image of the dorsal area of the retina is shown for (A) untreated, (B) NMDA and (C) NMDA+TUDCA experimental groups. High magnification images (D-I) correspond to central (D-F) and peripheral (G-I) areas of the retina for the three experimental groups. Scale bar 1 mm (A-C), 50 μm (D-I).

**Fig 5:**
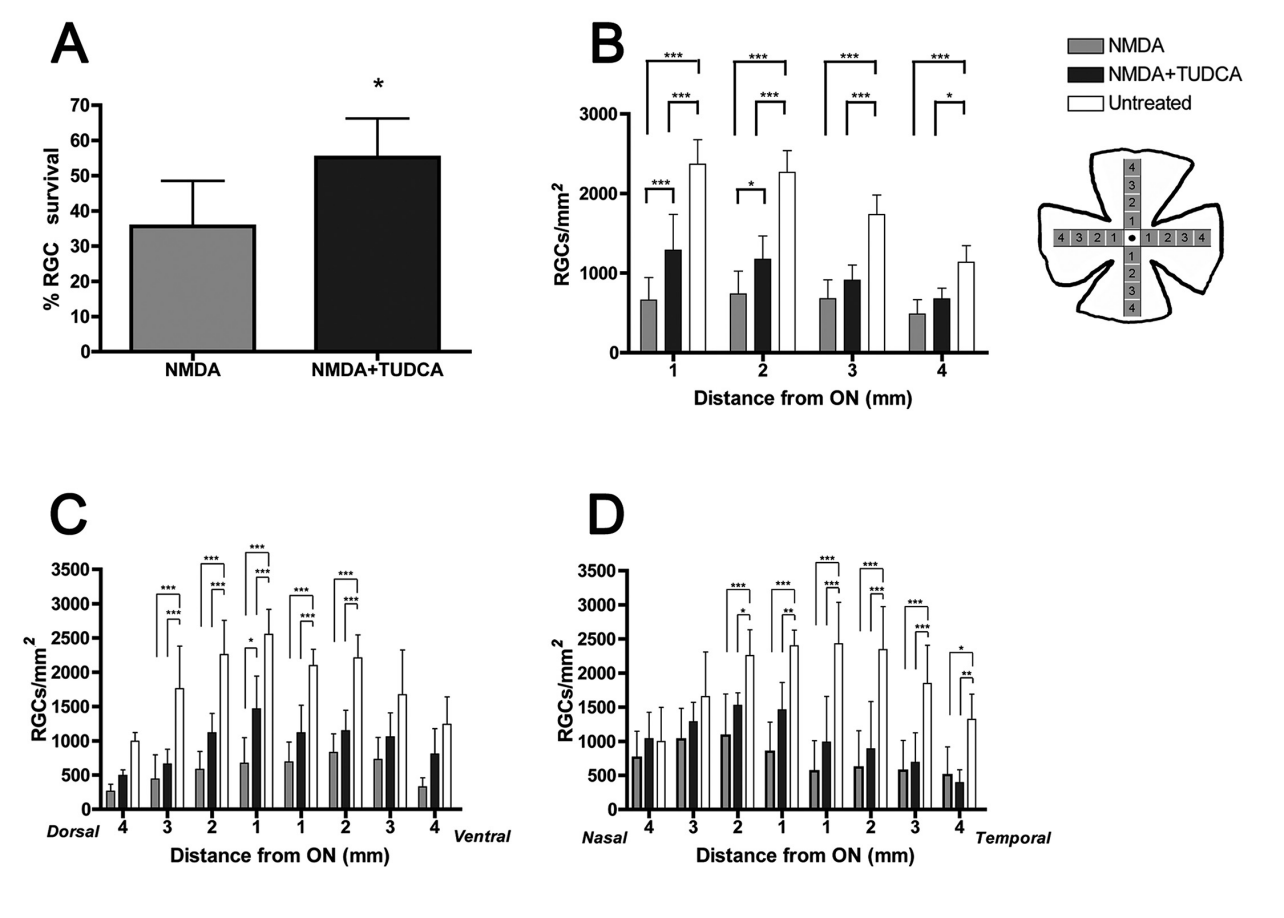
Quantitative analysis of RGC survival after NMDA-induced damage in the presence or absence of TUDCA. (A) Percentage (mean ± SD) of surviving RGCs with respect to control (untreated, 100%), in NMDA-injected retinas of animals treated with vehicle or TUDCA. *P<0.05; Student’s t-test. (B) RGC density was determined in 16 regions of interest at 4 different eccentricities along the dorsal-ventral and nasal-temporal axes of the retina, as represented schematically in the picture, and the data were plotted as an average (mean ± SD) of the 4 values corresponding to each eccentricity. (C) RGC density (mean ± SD) in each of the 8 regions of interest along the dorsal-ventral axis of the retina. (D) RGC density (mean ± SD) in each of the 8 regions of interest along the nasal-temporal axis of the retina. Asterisks in (B-D) indicate statistical significance *P<0.05, **P<0.01 and ***P<0.001; two-way ANOVA. In all cases (A-D) RGC density was determined in a total of 6 rats per experimental group (n = 6).

After evaluation of retinal function impairment by NMDA administration, we sought to verify that our findings correlated with a decrease in the number of surviving RGCs. To meet that purpose, RGCs were doubly labeled with antibodies against specific RGC markers, the transcription factor Brn3a and the RNA binding protein RBPMS [29–31], and retinal flat-mounts were photographed under a confocal microscope along the dorsal-ventral and nasal-temporal axes (Fig 4). As previously described, Brn3a staining showed nuclear localization in RGCs [29], consistent with its role as a transcription factor. In contrast, RBPMS immunoreactivity was observed in the cytoplasm, as expected for a post-transcriptional regulatory protein [31]. In agreement with previous reports [31,32], RGC density was higher in the central retina (2370 ± 306 and 2267 ± 272 RGCs/mm^2^, at 1 and 2 mm from the optic nerve head, respectively) and declined to the periphery (1735 ± 245 and 1137 ± 210 RGCs/mm^2^, at 3 and 4 mm from the optic nerve head, respectively) (Figs 4A and 5B), being the highest density located in the dorsal quadrant of the retina, at 1 mm from the optic nerve head (Fig 5C). Injection of 60 nmol of NMDA into the vitreous chamber induced a global decrease in the density of RGCs compared to non-injected control eyes (64% less; Fig 5A), which can be readily observed in retinal micrographs (Fig 4B, 4E and 4H).

### TUDCA promotes RGC survival against NMDA-induced damage

To further confirm the protective effect of TUDCA on RGCs, the density of double positive Brn3a/RBPMS cells in the GCL of retinal flat-mounts was compared between vehicle- and TUDCA-treated rats. Overall RGC survival, expressed as percentage of the average RGC density in non-injected control rats, was significantly higher (Student´s t test, P < 0.05) in TUDCA-treated animals after NMDA insult (Fig 5A). When RGC eccentricity was taken into account, a significant (two-way ANOVA, P < 0.001) protective effect of TUDCA was detected in the central areas of the retina (at 1 and 2 mm from the optic nerve head), but not in the peripheral areas (at 3 and 4 mm from the optic disc) (Fig 5B). The average RGC density in 15 out of 16 regions analyzed was higher in the TUDCA-treated group than in the vehicle-treated (Fig 5C and 5E). However, this difference was significant (two-way ANOVA, P < 0.05) only in 1 region located at the dorsal quadrant of the retina, 1 mm from the optic nerve head (Fig 5C).

## Discussion

There is considerable evidence regarding the cytoprotective role of TUDCA, a hydrophilic bile acid, in experimental models of inherited or induced retinal degeneration [5–11]. However, none of these studies targets RGCs for neuroprotection despite the fact that glaucoma, an optic nerve disease characterized by the irreversible loss of RGCs, is the second leading cause of blindness worldwide [33]. In the present work, we used an NMDA-mediated neurotoxicity model combined with a functional and morphological evaluation of the retina to demonstrate a neuroprotective effect of TUDCA on RGCs *in vivo*. We showed that systemic administration of TUDCA not only attenuated the functional changes associated with NMDA-induced retinal damage in Sprague-Dawley rats, but also delayed RGCs loss.

NMDA receptor subunits in the rat retina have been immunohistochemically localized on RGCs and displaced amacrine cells in the ganglion cell layer, as well as in a subset of amacrine cells in the inner nuclear layer [34]. Although the NR1C2’ subunit has also been localized in the outer plexiform layer, specifically within rod and cone photoreceptor terminals [34], a functional mapping of the NMDA receptor-mediated drive, using the channel permeant indicator 1-amino-4-guanidoutane, found no evidence of functional NMDA receptors in photoreceptor cells [35]. Consequently, NMDA treatment results primarily in the degeneration of amacrine and RGCs, but does not directly affect other cells in retina such as photoreceptors. In fact, the presence of TUNEL positive profiles, a hallmark of apoptosis, has been detected essentially in the ganglion and inner cell layers of the retina 24 hours after intravitreal administration of NMDA [36–38]. As expected, when we injected NMDA into the vitreous chamber of Sprague-Dawley rats it caused a significant decrease in the number of RGCs, as visualized by immunohistochemistry with Brn3a and RBPMS antibodies, 72 hours after administration. Quantification of surviving RGCs evidenced that cell death was not homogeneous throughout the whole retina, but occurred mainly around the optic nerve head (central retina) and at the dorsal and temporal quadrants (Fig 5C and 5D). This could probably be attributable to a non-homogeneous distribution of NMDA when delivered into the vitreous chamber, so the more affected areas are those near the injection site.

Degeneration of RGCs and, presumably, amacrine cells would account for the reduction in the amplitude of several parameters of the dark-adapted ERG, including the pSTR, nSTR, b-wave and OPs, observed in the present study. However, we also detected a significant reduction in the amplitude of the scotopic a-wave, which mainly reflects the activity of photoreceptors. This is in agreement with other reports showing, as well, decreased amplitude of the scotopic a-wave following NMDA-induced retinal damage [36–39]. In a previous work, Bui and colleagues [24] reported that blockade of inner retinal activity by intravitreal injection of 0.8 mM NMDA did not alter the amplitude of the scotopic pSTR, a-wave or b-wave of the rat full-field ERG. Importantly, this experiment was designed to suppress light responses of third-order retinal neurons by depolarizing their membranes, and ERG recording was carried out as short as 30 minutes after NMDA administration. Therefore, long-term effects of retinal exposure to NMDA were not evaluated [24]. A possible explanation for the decrease in the a-wave amplitude observed in this and other studies is that NMDA is incidentally inducing functional impairment of photoreceptors. Overstimulation of NMDA receptors has been demonstrated to cause activation of NOX2, an enzyme that generates superoxide, producing oxidative stress in neighboring cells [20,40]. Likewise, the increase in pro-inflammatory cytokines in the retina and the recruitment/activation of microglia are two factors linked to NMDA-mediated neurotoxicity that may help propagate the damage to other retinal cells [41,42].

Overactivation of NMDA receptors triggers neuronal toxicity and degeneration. A pharmacological approach for the treatment of glaucoma with NMDA receptor antagonists has been proposed [43]. However, NMDA receptors mediate synaptic transmission and plasticity, which are essential for the normal function of the nervous system. Moreover, their activity is coupled to the transcriptional control of the glutathione biosynthesis, important for the maintenance of the cellular redox balance [44]. Thus, NMDA receptor complete blockade may imply intolerable side effects. An interesting alternative would be the use of antiapoptotic compounds as RGC neuroprotectans. One of such compounds is TUDCA, whose antiapoptotic activity has been previously demonstrated in photoreceptor degenerations. In the present study we demonstrate TUDCA improves RGC survival and function following excitotoxic insult. The detailed molecular mechanisms that mediate TUDCA protection have not been fully investigated, though it seems to block apoptosis at various levels, including the alleviation of endoplasmic reticulum stress [45], the stimulation of the PI3K and MAPK (p38, ERK1/2) survival pathways [46] and the blockade of Bax translocation to the mitochondria impeding subsequent cytochrome c release [47]. Specifically in the retina, TUDCA has been shown to attenuate oxidative stress in photoreceptor degenerations [7,10] and to restrain neuroinflammation, preventing the detrimental effects of uncontrolled microglia activation [48,49]. The ability to interfere with multiple death mechanisms may underlie the neuroprotective action of TUDCA on RGCs. In summary, we demonstrate that systemic administration of the antiapoptotic TUDCA attenuates apoptosis and improves functionality of RGCs.
